# Hypertriglyceridemia induced by aripiprazol: about a clinical case

**DOI:** 10.1192/j.eurpsy.2022.1877

**Published:** 2022-09-01

**Authors:** G. Jordán, A. San Miguel, J. García Jimenez

**Affiliations:** AGS Sur de Granada (Motril), Salud Mental Ags Sur Granada, Granada, Spain

**Keywords:** metabolic syndrome, Hypertriglyceridemia, Aripiprazol

## Abstract

**Introduction:**

41 years-old man diagnosed of schizophrenia and peripheral spondyloarthropathy HLA-B27 (-) in treatment with methotrexate. Psychiatric background: First psychotic episode at 18, with no further medical monitoring. In 2018 he underwent a new episode consisting in auditory hallucinations, delusional ideas and clinophilia of months of evolution. He was sent to a Psychiatric Rehabilitation Unit and prescribed aripiprazole 20mg. The routine blood analysis revealed triglycerides level of 414mg/dL, with previous normal levels (123 mg/dL), without no other cause to justify it.

**Objectives:**

To study the relationship between aripiprazole treatment and acute hypertriglyceridemia.

**Methods:**

A clinical case is presented and available bibliography about the relation between aripiprazole and acute hypertriglyceridemia is reviewed.

**Results:**

Hypertriglyceridemia was confirmed in the second analysis, so we concluded it was due to the start of aripiprazole, after rejecting other potential causes. Aripiprazole was replaced by cariprazine 3mg because of its similar profile. The analysis was repeated after a month and the normalization of the triglyceridemia (159mg/dL) was verified, while cholesterol levels remain stable. Moreover, the patient experienced an improvement in akathisia and sedation.

**Conclusions:**

Although metabolic impact is not expected with aripiprazole, after reviewing the bibliography we have found a clinical trial and a case series that described this adverse effect. Our case highlights the importance of closely monitoring of patients in whom an antipsychotic treatment is started due to the high mortality and morbidity related to cardiovascular diseases.

**Disclosure:**

No significant relationships.

